# Fate of Dissolved Methane from Ocean Floor Seeps

**DOI:** 10.1021/acs.est.5c03297

**Published:** 2025-04-23

**Authors:** Tor Nordam, Anusha L. Dissanayake, Odd Gunnar Brakstad, Sigrid Hakvåg, Ida Beathe Øverjordet, Emma Litzler, Raymond Nepstad, Annika Drews, Johannes Röhrs

**Affiliations:** † SINTEF Ocean, 7010 Trondheim, Norway; ‡ Department of Physics, Norwegian University of Science and Technology, 7491 Trondheim, Norway; § Independent Researcher, EnvSoln, Badulla, Uva Province 90000, Sri Lanka; ∥ Formerly at SINTEF Ocean, 7010 Trondheim, Norway; ⊥ Now at Landeskreditbank Baden-Württemberg - Förderbank, 70174 Stuttgart, Germany; # Norwegian Meteorological Institute, 0371 Oslo, Norway

**Keywords:** methane, seafloor seeps, mass transfer, vertical mixing, climate

## Abstract

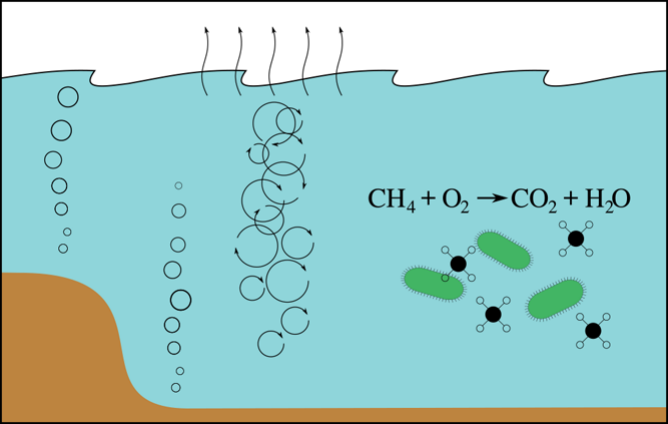

Methane is an important greenhouse gas, with a global warming potential that is far higher than that of CO_2_. Methane from seafloor seeps, whether naturally occurring or in relation to petroleum infrastructure, has been suggested to be a significant contribution to greenhouse gas releases. Here, we consider the fate of methane from seeps on the Norwegian continental shelf by means of models for dissolution of methane from rising bubbles, mixing and biodegradation of dissolved methane, and mass transfer to the atmosphere. Laboratory experiments with tritium-labeled methane have been conducted to help determine the biodegradation rate of methane in natural seawater, and the results, together with literature data, have been used to guide the modeling. From the modeling study, we present results as a function of biodegradation half-life, treating this as a free parameter to reflect the considerable span in values reported in the literature. Considering three different locations on the Norwegian continental shelf, we find that if the biodegradation half-life of methane is in the range of a 9 to 16 days, as suggested by our experiments, then about 57–68% of the released methane will biodegrade in the water column from a seep at 65 m depth. For deeper locations of 106 and 303 m, we find respectively 75–83%, and more than 99% biodegradation.

## Abstract 2

Methane seeps may be an important source of greenhouse gas. We use experimental work to help determine biodegradation rates of methane in seawater, and models to study the fate of methane, in particular the fraction that reaches the atmosphere.

## 1 Introduction

Methane is one of the most abundant and most potent greenhouse gases with a global warming potential (GWP) of 79.7 times that of CO_2_ over 20 years, and 27 times over 100 years (for nonfossil methane, see, e.g., Table 7.15 in Forster et al.^1^). Its atmospheric concentration is rising at an accelerating rate.^1,2^ Methane formed in marine sediments can escape and form sea floor seeps, and seeps can also occur due to anthropogenic activity, in addition to methane escaping from deeper gas reservoirs.^3,4^ Both thermogenic and biogenic processes are responsible for the generation of methane in the marine environment. While deep methane sources are the sources for thermogenic formation, methane is also formed in deep anoxic sediments from organic matter or carbon dioxide by methanogenic microbes.^5^ Methane from such gas accumulations can be released by human intervention, e.g., leaking out through and along petroleum wells.^6−8^

The fate of methane released from the sea floor is key to estimating the potential climate impact. Marine seeps potentially account for approximately 20 × 10^12^ g methane per year, i.e., 4% of the global emissions of methane to the atmosphere.^9^ An approximate amount of 1.8 × 10^18^ g methane is also expected to exist trapped in gas hydrates in terrestrial permafrost and marine environments, with 99% in marine sediments.^10−12^

Rising seawater temperature may lead to a dissociation of methane gas hydrates from the sediments, which is of particular future concern in shallow Arctic oceans. One example of a region with natural seeps and gas emission clusters is in the Arctic around Svalbard,^13−15^ where methane gas flares have been recorded southwest of Spitsbergen, on the west Spitsbergen continental margin, and in Nordfjorden. Methane in this region is primarily of biogenic origin,^15^ and parts of it are thought to be released as the gas hydrate stability zone moves to greater depths due to increasing temperatures.^16^

The purpose of this paper is to investigate the fate of methane released in seafloor seeps on the Norwegian continental shelf (NCS). Once a methane bubble is released from a seep at the sea floor, methane begins dissolving into ambient water while the bubble rises. In shallow water, some fraction of the methane will reach the atmosphere directly with the bubbles, while in deeper locations, all or most of the methane might dissolve in the water. Methane bubbles from seeps can be observed with acoustic methods, and in several studies the acoustic signal from the bubbles has been found to disappear within 100–150 m from the sea floor, indicating that the bubbles may have dissolved completely.^17,18^

The dissolved methane will then be mixed in the water column, partially oxidized into CO_2_ and water by methanotrophic bacteria, and may partially escape into the atmosphere through mass transfer at the air-sea interface.^19,20^ Earlier studies^6,8^ have also discussed the split between direct bubble transport and dissolution, but have assumed biodegradation to be very slow, which would imply that all or most of the dissolved methane eventually reaches the atmosphere. Available literature data on methane biodegradation rates in seawater shows significant variations, with half-lives ranging from a few days^21−23^ to weeks^15^ and even several years.^21,24,25^ This variation has implications for modeling the fate of methane in the water column and subsequently for determining fluxes of methane into the atmosphere. Laboratory experiments with tritium-labeled methane have therefore been performed to help determine the biodegradation rate of methane in seawater.

In the current study, we explicitly include methane degradation half-life as a free parameter in our model and discuss its importance for the results. We consider three different locations on the NCS, with depths of 65, 106, and 303 m. We use a full year of modeled, depth-resolved temperature, salinity, and vertical diffusivity data for each station, both to highlight seasonal variations and to obtain annual averages. While we focus on depths that are relevant for the NCS, we note that in very deep waters (deeper than 500–1000 m, depending on location), the time scale for dissolved gases to escape to the atmosphere may be hundreds of years or longer.^26^

**Figure 1:**
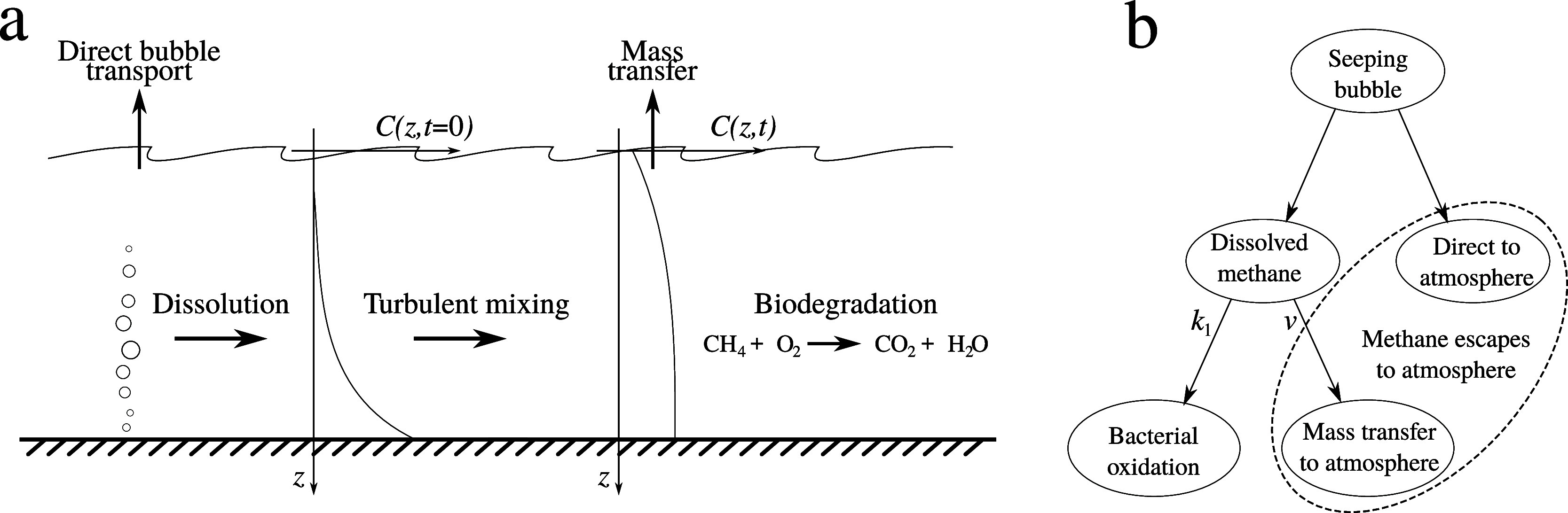
(a) Illustration of the fate of methane after release of bubbles at the sea floor. (b) Schematic illustration of the different pathways that methane from seeps can take. The branching parameters *k*_1_ and *v* are discussed in Section 3.5.

The fraction of methane released from seeps, which ultimately ends up in the atmosphere, is thus a balance between different pathways: First, there is a balance between direct bubble transport of methane to the surface versus dissolution in the water column. Second, there is a balance between escape to the atmosphere via mass transfer, or biodegradation of dissolved methane to CO_2_ and water. The different fate processes are sketched in Figure 1a, and the pathways are shown schematically in Figure 1b. We note that oxidation of methane into CO_2_ gives a lower climate impact, given the lower GWP of CO_2_ in the 20–100 year time frame.^1^ A thorough understanding and quantification of the pathways of seeped methane are hence needed for the estimation of the climate impact of methane from seeps.

While the conversion of methane to CO_2_ in the ocean will reduce the ability of the ocean to absorb CO_2_, we do not attempt to quantify this other than to note that it is likely to be a small effect. The ocean is estimated to absorb around 2.6 × 10^15^ g/year of anthropogenic carbon (in the form of CO_2_) from the atmosphere,^27^ while the estimated amount of methane entering the oceans from seeps is estimated at 30 × 10^12^–50 × 10^12^ g/year.^28^ If we assume that CO_2_ from methane degradation replaces CO_2_ absorbed from the atmosphere at a 1:1 ratio, this would mean a 1–2% reduction in the amount of CO_2_ absorbed from the atmosphere.

## 2 Materials and Methods

The current study combines both experimental and modeling work, where the experimental work provides input to the model. Below, we first present the experimental work (Section 2.1) and the Bayesian approach (Section 2.2) which we use to find biodegradation rates of methane in seawater. Then we present a set of numerical models that capture the relevant mechanisms shown in Figure 1: Bubble rise and dissolution (Section 2.3), vertical turbulent mixing (Section 2.4), biodegradation, and mass transfer to the atmosphere (Section 2.5).

### 2.1 Experimental Determination of Biodegradation Rates

The biodegradation rate of dissolved methane in seawater is an important parameter in a fate model for methane from seeps, and thus, we have performed a set of experiments measuring the rate of biodegradation of methane in natural seawater from a Norwegian fjord, using isotopically labeled methane. The experiments were performed by spiking tritium-labeled methane (^3^H-CH_4_) to natural seawater preadapted with regular CH_4_ in sealed flasks and determining ^3^H-H_2_O production after an incubation period at 5 or 8.5 °C, assuming first-order kinetics. A description is given below, with additional details available in a technical report.^29^

#### 2.1.1 Seawater and Methane

Natural subsurface seawater (SW) used in the studies was provided from an intake at 80 m depth in the Trondheimsfjord (63°26′N, 10°24′E). The SW enters the laboratory of SINTEF Ocean through a pipeline system and passes through a sand filter to remove coarse particles. The depth of the inlet is well below the thermocline, securing a stable temperature of 6–8 °C and 34 ppt salinity throughout the year.^30^ Mineral nutrient analysis has previously shown concentrations of 19 μg/L total P, 16 μg/L o-PO_4_-P, 130 μg/L NO_2_ + NO_3_-N, and 3 μg/L NH_4_-N, and less than 0.05 mg/L Fe,^31^ as well as approximately 8 mg/L dissolved oxygen.

High quality (HiQ) CH_4_ gas (purity greater than 99.5%) was purchased from the Linde Gases Division (Pullach, Germany). Tritium-labeled methane was purchased from American Radiolabeled Chemicals, Inc. (St. Louis, MO, USA), provided as gas in break-seal ampules of 1 mCi (specific activity 15 Ci/mmol).

#### 2.1.2 Ex Situ Biodegradation Experiments

The stock solution of HiQ CH_4_ was prepared in 100 mL crimp-sealed serum flasks filled with SW. HiQ CH_4_ gas was bubbled into the SW for 120 s. (Methods for preparation and analyses have previously been described.^22^) Crimp-sealed flasks of test, control, and blank solutions (100 mL) were completely filled with natural SW. CH_4_ stock solution was added to the test and control flasks to achieve CH_4_ concentrations of 6 μmol/L, as measured by GC-FID (gas chromatograph with flame-ionization detector). The control flasks were sterilized (100 mg/L of HgCl_2_). The test, control, and blank solutions were incubated (upside down to avoid gas leakage) at 5 °C for 7 days for pre-adaptation of the seawater inoculum in the test flasks. All incubations were performed in the dark, with no stirring.

Experiments for studying methane oxidation with ^3^H-CH_4_ were performed mainly as described previously.^25,32,33^ Each aliquot of ^3^H-CH_4_ gas was dissolved in 5 mL of hexane to make stock solution, and 50 μL of the ^3^H-CH_4_ stock solution was applied to each crimp-sealed serum flask with SW test or control solution (nominal concentration 6.5 nmol/L ^3^H-CH_4_). Some samples were sacrificed to determine the radioactivity before incubation. Multiple replicates of all treatments were incubated at 5 or 8.5 °C.

Sampling was performed by removing most of the liquid, leaving 10 mL in the flasks for liquid analysis of the formed oxidation product (^3^H-H_2_O). These 10 mL volumes in the test and control solutions were sparged with N_2_ gas for 2 h to remove residual ^3^H-CH_4_ in the samples. All test, control, and blank solutions were then analyzed by applying 1 mL of the sample in 10 mL of scintillation cocktail (Ultima Gold scintillation cocktail; PerkinElmer, Inc.) in 20 mL scintillation vials and measuring radioactivity in an LS 6500 multipurpose scintillation counter (Beckman Coulter, Inc.). The results were recorded as disintegrations per minute.

### 2.2 Bayesian Analysis

We use statistical parameter estimation and Bayesian analysis^34−37^ to extract as much useful information from the experimental data as possible, including uncertainties in the estimated parameter values. Below, we briefly restate the key experimental steps and parameters relevant to formulating a generating model.

- Tritium-labeled CH_4_ is added to seawater. Total activity prior to incubation, *C*_tot_, is measured.
- Sterile controls are sparged with N_2_ to remove labeled CH_4_, and the remaining activity is measured, representing the “background” activity, *C*_b_.
- Samples are incubated for different numbers of days (2, 4, 13, 17, 27), at different temperatures (5, 8.5 °C), then sparged with N_2_, and activity is measured. *C*(*t*) is the activity after incubation for time *t* and sparging.

Given the assumption of first-order decay, we propose a generating model for the data of the exponential decay type with a concentration-independent half-life *t*_1/2_:
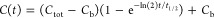
1This model assumes that the activity in the sparged tests at *t* = 0 is equal to the background (*C*_b_), increasing asymptotically toward *C*_tot_ as an increasing fraction of the labeled methane is converted into labeled water. In line with earlier work, we assume no lag phase.^22^

In order to make use of data collected at different temperatures *T*, a *Q*_10_ scaling^38,39^ of the half-life was used,
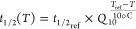
2where *t*_1/2_ref__ is the half-life at temperature *T*_ref_ (in our case, *T*_ref_ = 5 °C), and we use a constant value of *Q*_10_ = 2, which has been used elsewhere in the literature for oxidation of both methane and other light hydrocarbons in seawater.^38−40^*Q*_10_ = 2 means that an increase in temperature by 10 °C leads to a halving of the half-life (doubling of the rate).

By using the available data and fitting to eq 1, we estimate the optimal parameters (*t*_1/2_, *C*_b_, *C*_tot_) in two different ways: by minimization of the negative log-likelihood function (using the Python package lmfit(41)), and by Markov-Chain Monte Carlo (MCMC) sampling of the implicitly defined posterior (using the Python package emcee(37)). The latter is also used to calculate the uncertainty bounds. The likelihood function is the log of the probability of the data, given the (true) parameters:^36,37^
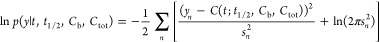
3where the data uncertainty *s*_*n*_ = e^lnσ^ is estimated as a single positive value (σ) together with the model parameters. We used uniform priors for the parameter values: For *C*_b_ and *C*_tot_, we require only that they are non-negative, while we assumed the half-life, *t*_1/2_, to be between 0.25 days and 100 days. The results are listed in Section 3.1

### 2.3 Single Bubble Model

While rising through the water column, the size and shape of a bubble evolve as it expands due to an ambient pressure drop and mass exchange with ambient water. Mass exchange involves the dissolution of methane into seawater, and dissolved gases in the seawater, mainly nitrogen and oxygen, entering the bubble.^17^

In this study, we use the Single Bubble Model (SBM) from TAMOC to track individual bubbles in the water column, model the gas exchange between the bubble and the ambient, and calculate direct bubble transfer of methane to the surface. TAMOC is an open source modeling suite^1^ for subsea oil and gas releases, and has been extensively validated against both laboratory and field data.^42−47^ The SBM can take into account nonideal gas behavior, mass and heat exchange with the ambient environment, as well as effects of hydrate formation, and has been used in previous studies to predict the behavior of sea floor seeps of methane, carbon dioxide, etc.^48−54^

The density of the bubble evolves according to the composition, pressure, and temperature, based on the thermodynamics of the gas mixture estimated by the Peng–Robinson equation of state.^55,56^ A volume translation parameter is used to correct the density from the Peng–Robinson equation.^56,57^ The aqueous solubility of each gas component in the bubble under local conditions is estimated using the modified Henry’s law and mixture fugacities.^17,56,58^

The terminal rise velocity in the model varies according to the size and shape of the bubble, viscosity, interfacial tension, and density difference between the bubble and ambient water. Bubbles can be defined into different groups, namely, spherical, spherical-cap or ellipsoidal shape based on their size and certain nondimensional numbers.^44,59,60^ The bubble–water mass transfer coefficient also depends on these shape categories^59,61^ and on the presence of a surfactant film on the bubble surface, which may obstruct mass transfer.^59^ Formation of gas hydrate shells can also reduce mass transfer across the bubble surface^44,54,62,63^ and lead to unusual bubble shapes.^64^ We note that in this work, we consider releases at depths and temperatures where we can ignore hydrate formation.

### 2.4 Turbulence Modeling

Eddy diffusivity is a parametrization of the effective mixing caused by turbulence and cannot be measured directly but must be inferred from other measurements or obtained from a model. Even though the eddy diffusivity does not directly correspond to an actual physical quantity, it is typically used in models to calculate the diffusion of scalar properties such as salinity and concentration of other dissolved chemicals.

Here, the vertical eddy diffusivity has been modeled using GOTM^65^ (version 6.0). GOTM solves a prognostic equation for turbulent kinetic energy (TKE) and a turbulent length scale in the water column and thereby predicts an effective turbulent exchange rate, i.e., diffusivity, *K* (see eq 4). The prognostic equation models the evolution of TKE as a result of shear production of turbulence due to currents and wind stress, buoyancy production, and turbulent dissipation. The model also accounts for the inertia of turbulence through a tendency term and the inhibition of turbulence due to stable stratification.

**Figure 2:**
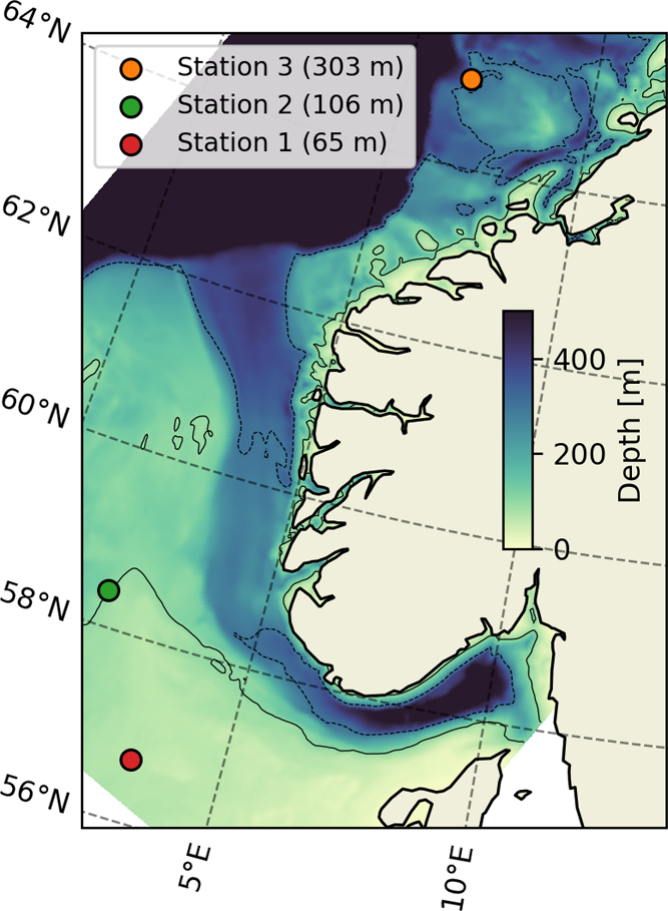
Locations considered in the modeling. The locations were chosen to represent a range of depths typical for NCS. The 100 m (continuous line) and 300 m (dashed line) depth contours are shown.

Water column hydrography (temperature, salinity, and horizontal ocean currents) is taken from a three-dimensional regional ocean model (NorShelf^66^), and provided as input to GOTM, using a relaxation toward the input. For the upper boundary condition for TKE and the generic length scale for GOTM, we used atmospheric forcing data from ERA5.^67^ We ran GOTM for three different locations on the NCS, generating diffusivity profiles for the whole year of 2019. The GOTM configurations applied in this study are modified from the example case “NNS annual” provided by the developers of GOTM.^68^ Our setup for the three stations can be found on GitHub^2^, and the locations are shown on a map in Figure 2.

**Figure 3:**
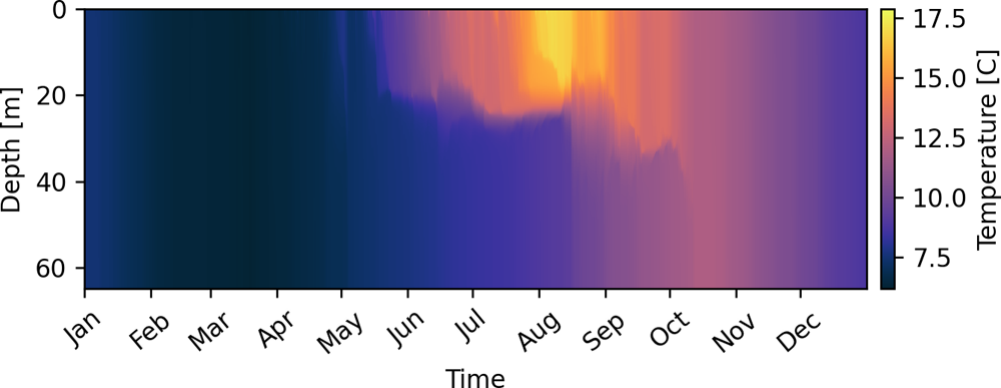
Temperature for Station 1, for the year of 2019.

As an illustrative example, we show the temperature for 2019 at Station 1 (65 m depth) in Figure 3. We clearly see that the water column is well mixed at the beginning of the year, with constant temperature throughout. Starting from May, a seasonal pycnocline develops from surface heating (and also fresher water from the Norwegian coastal current^69^) and divides the water column into a surface mixed layer and a deep interior. This division lasts until the autumn, when the surface mixed layer deepens as a result of wind-induced mixing and surface cooling, and eventually the water column is again homogeneous from late October and into winter. The pycnocline suppresses exchange between the surface mixed layer and the deeper waters from around May to October.

Additional data for all three stations are shown in the Supporting Information (Section S3). The same general observations as above also apply to Station 2 (106 m depth), while the water column is never completely homogeneous at Station 3 (303 m depth).

### 2.5 Diffusion-Reaction Solver

The transport of the dissolved methane was modeled with the diffusion-reaction equation, accounting for vertical mixing, biodegradation, and mass transfer to the atmosphere. The one-dimensional diffusion-reaction equation, describing the time development of a concentration, *C*(*z*,*t*), is given by
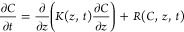
4where *K*(*z*,*t*) is the diffusivity, and *R*(*C*,*z*,*t*) is a reaction term.^70^ In the current study, the only reaction considered is the first-order decay of methane with a fixed rate coefficient, meaning that the reaction term takes the form *R* = −*k*_1_*C*, where *k*_1_ is the concentration-independent rate coefficient. We note that the relation between the rate coefficient and the half-life is *k*_1_ = ln(2)/*t*_1/2_.

The diffusivity represents the mixing caused by the combination of turbulence and molecular diffusion in the water column (see, e.g., pp. 20–21 in Thorpe^71^). It is generally a function of both depth and time and is strongly linked to the water column stratification. A stably stratified water column will have limited exchange between adjacent layers of water, while a homogeneous water column admits a larger eddy motion in the vertical direction, leading to faster mixing.

As initial conditions for the diffusion-reaction model, output from TAMOC is used to find a concentration profile of deposited dissolved methane. At the sea floor, we assumed a zero-flux boundary condition, while at the air-sea interface, we used a prescribed-flux boundary condition, determined by the concentration of dissolved methane in the surface layer and the mass transfer coefficient. The mass-transfer flux, *j*, across the surface is given by
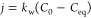
5where *k*_w_ is the mass transfer coefficient, *C*_0_ is the concentration of methane in the surface water, and *C*_eq_ is the concentration at which the methane in the surface water would be in equilibrium with the partial pressure of methane in the atmosphere. For the purposes of this study, we simply let *C*_eq_ = 0. This means that we assume that the partial pressure of methane in the atmosphere is too small to have a retarding effect on the escape of dissolved methane from the water column.

It is common to parametrize the mass transfer coefficient at the sea surface in terms of the wind speed.^72,73^ In the current study, we use the parametrization^73^
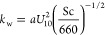
6where *a* = 6.97 × 10^–7^ s/m is an empirical parameter, *U*_10_ is the wind speed at 10 m, and Sc = ν/*D* is the Schmidt number, giving the ratio between the kinematic viscosity of seawater and the molecular diffusivity of methane dissolved in seawater. We have used Sc = 677.^74^

The diffusion-reaction equation was solved numerically with a finite-volume method, using the Crank-Nicolson scheme, which was found to give second-order accuracy in space and time,^75,76^ and conservation of mass to around 10 significant digits. Further details of the numerical solver are found in the Supplementary (Section S2), and the code is available on GitHub^3^.

### 2.6 Modeling Inputs

The main inputs to the model are seabed depth, bubble size (equivalent diameter) and composition, methane biodegradation rate, ambient profiles of salinity, temperature, dissolved gases, and vertical diffusivity. The different inputs and their sources are briefly summarized in Table 1.

**Table 1:** Summary of the Input to the Bubble Model (SBM), the Turbulence Model (GOTM), and the Diffusion-Reaction Model (DR)

model	input variable	units	comment
All	depth	m	three values chosen, 65, 106, 303 m, representative of NCS.
SBM	initial size	mm	4.5 mm, chosen based on reported observations.^8^
SBM	bubble composition		assumed pure methane from seep.
SBM	temperature	°C	profiles taken from NorShelf.^66^
SBM	salinity	ppt	profiles taken from NorShelf.^66^
SBM	dissolved N_2_ and O_2_	kg/m^3^	calculated from assumption of equilibrium with atmosphere.
GOTM	temperature	°C	timeseries of profiles taken from NorShelf.^66^
GOTM	salinity	ppt	timeseries of profiles taken from NorShelf.^66^
GOTM	wind speed	m/s	timeseries taken from ERA5.^67^
GOTM	atmosphere data		temperature, pressure, humidity, cloud cover. Timeseries from ERA5.^67^
DR	Eddy diffusivity	m^2^/s	timeseries of profiles, output from GOTM.
DR	biodegradation half-life	days	range of 1–1000 days, based on literature and own experiments.
DR	wind speed	m/s	time series taken from ERA5.^67^
DR	mass transfer coefficient	m/s	parameterised from wind speed by eq 6.^73^

## 3 Results and Discussion

In Section 3.1, we present the results of the biodegradation experiments. Then, in Section 3.2, we show some example results from the single bubble model in isolation, before we put everything together and present results from the full modeling chain: First as results of single runs (Section 3.3), to introduce the type of results we obtain from the modeling, and then as both time-resolved and averaged results over a full year (Section 3.4). In Sections 3.5 and 3.6, we discuss the main drivers of uncertainty in the results, and finally, in Section 3.7, we present some closing remarks.

### 3.1 Experimental Results

**Figure 4:**
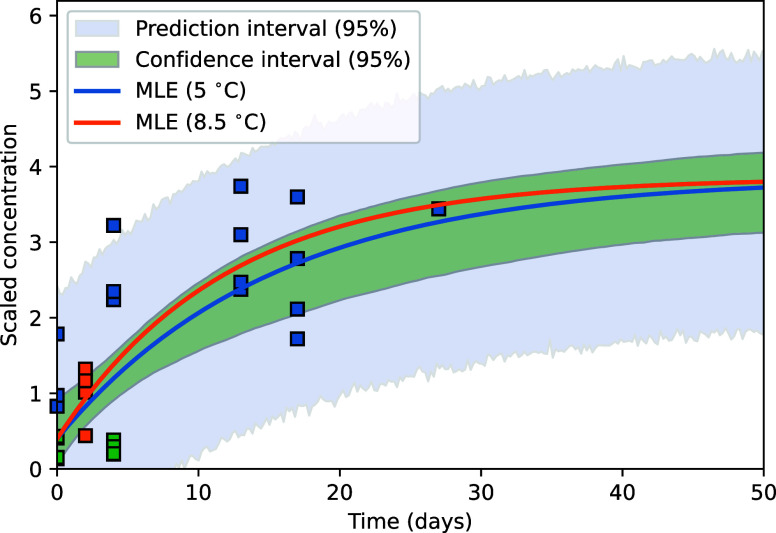
Normalized concentration of labeled water as measured in the lab, and best fit to the data, corresponding to a half-life of *t*_1/2_(5 °C) = 10.6 days (blue line). The blue and green markers are two sets of experiments performed at 5 °C, while the orange markers show an experiment done at 8.5 °C.

The results of the maximum likelihood estimation (MLE) and MCMC analysis are shown in Figure 4. We note that all concentrations have been normalized with the average of *C*_b_, which does not affect the fitted half-life. Estimated parameter values with uncertainties are shown in Table 2, with additional details found in the Supporting Information (Section S1). Rounding to whole days, we find that based on these experiments, *t*_1/2_ is most likely in the range 9–16 days (the 16–84 percentile range). This is broadly in line with, e.g., a study near seep sites in the Barents Sea that found first-order rate coefficients of 0.035–0.072 d^–1^, corresponding to half-lives of 10–20 days,^15^ although concentrations were somewhat lower in those cases.

**Table 2:** Summary of the MLE and MCMC Analysis^a^

parameter	unit	MLE value	posterior median	MLE standard error	posterior 16-percentile	posterior 84-percentile
*C*_tot_		3.853	3.883	0.299	3.588	4.184
*t*_1/2_	days	10.642	11.622	3.345	8.904	15.557
*C*_b_		0.407	0.461	0.231	0.237	0.696
lnσ		–0.185	–0.133	0.112	–0.241	–0.017

a The concentrations are normalized and thus dimensionless.

### 3.2 Single Bubble Model Results

As an illustrative example, we run the SBM to study the fate of a single bubble of pure methane, initially released from a 65 m depth at Station 1 with a diameter of 4.5 mm. Temperature and salinity profiles were taken from NorShelf,^66^ and ambient profiles of dissolved oxygen and nitrogen were calculated by TAMOC, based on the assumption of equilibrium with the atmosphere. We have assumed the initial concentration of methane in water to be zero, and we have assumed a “clean bubble” mass transfer coefficient. In reality, there might be some dissolved methane present in the water column initially, especially in a seep area, and surfactants in the water might stick to the bubble and make it “dirty”.^77^ Both of these effects would lead to slower dissolution of methane and increased direct bubble transport to the atmosphere, but we have made these assumptions, as we are mainly interested in the eventual fate of the methane that dissolves into the water column.

**Figure 5:**
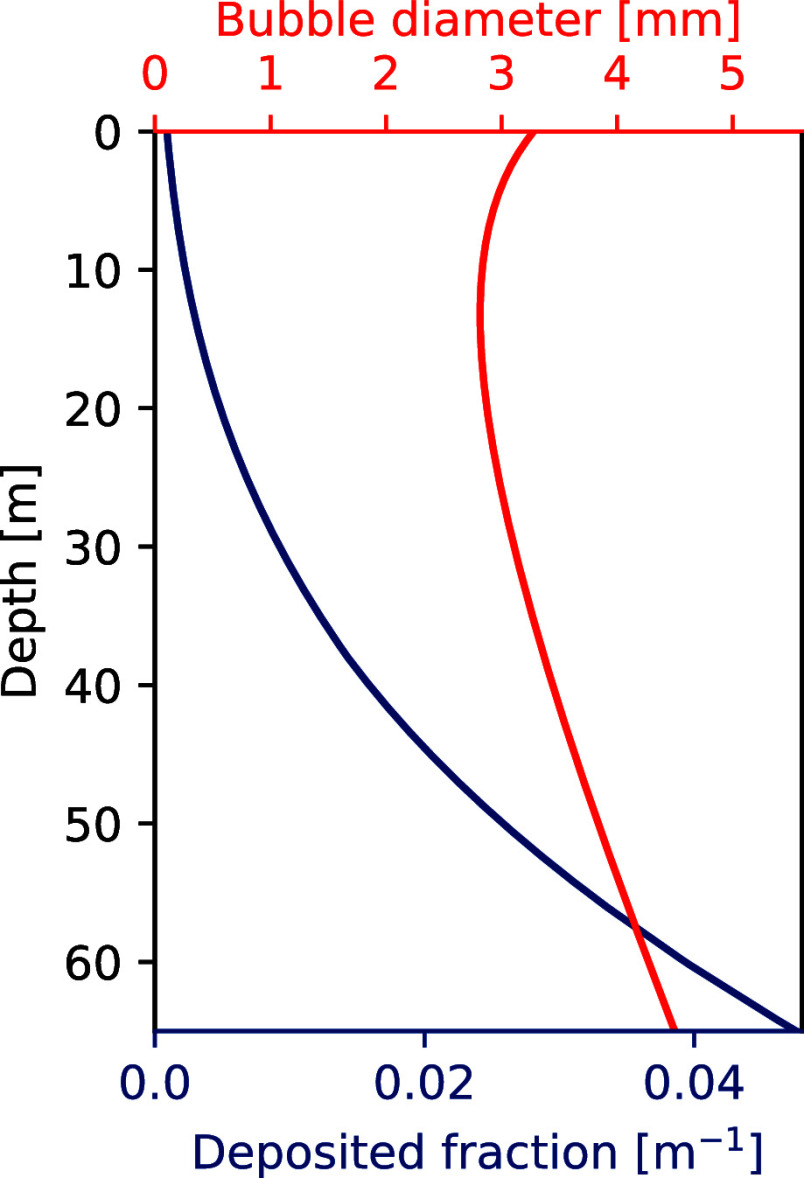
Results of running the SBM for a 4.5 mm methane bubble released at a 65 m depth at Station 1. The red line (top axis) shows the size of the bubble, and the blue line (bottom axis) shows the fraction of the original methane deposited per meter.

The SBM calculates the bubble diameter and the fraction of methane remaining in the bubble, and from the latter, we infer the fraction of the original methane in the bubble deposited per meter of water column, as shown in Figure 5. The rise velocities of the bubbles vary with depth and case, but were for the most part between 0.25 and 0.35 m/s. Modeled bubble shapes were ellipsoid in all cases. Additional results for the single bubble model, including rise velocity as a function of depth, are shown in the Supplementary Section S4.

For small seeps, where the bubbles may be assumed to behave independently, we assume that the deposited fraction remains the same for all bubbles of the same initial size. Note that in this case, most of the methane (around 99%) is dissolved into the water column before the bubble reaches the surface, and when the bubble reaches the surface, it consists mainly of nitrogen and oxygen. This gas exchange has also been observed in the laboratory,^78^ and our results are also broadly in agreement with modeling shown in, e.g., von Deimling et al.^8^ (see their Figure 14), who find that a 5 mm bubble released from 72 m depth will reach the surface, but with a very small fraction of methane left.

### 3.3 Diffusion-Reaction Model: Single Runs

The SBM shows that most of the methane from seeps dissolves in the water column. To study the fate of the dissolved methane, the deposition as a function of depth (Figure 5) is used as an initial condition for the diffusion-reaction model. Additionally, the model takes as input eddy diffusivity and wind speed (from which the mass transfer coefficient at the sea surface is estimated). A subtle point is that we do not, in fact, work with physical concentrations of methane but with fractions. From the SBM, we calculate the fraction of the mass in the bubble dissolved per depth, and we use this as input to the diffusion-reaction model to calculate what fraction of the mass is biodegraded and what fraction reaches the atmosphere. We assume that the bubbles are independent and the same fractions hold for all bubbles. The rate of methane released to the atmosphere can then be found by multiplying the release rate of a seep with the fraction found from the modeling. The approach of using fractions instead of concentrations is justified, as the diffusion-reaction equation is linear in concentration, which means that scaling the initial condition leaves the ratio between outcomes unchanged.

**Figure 6:**
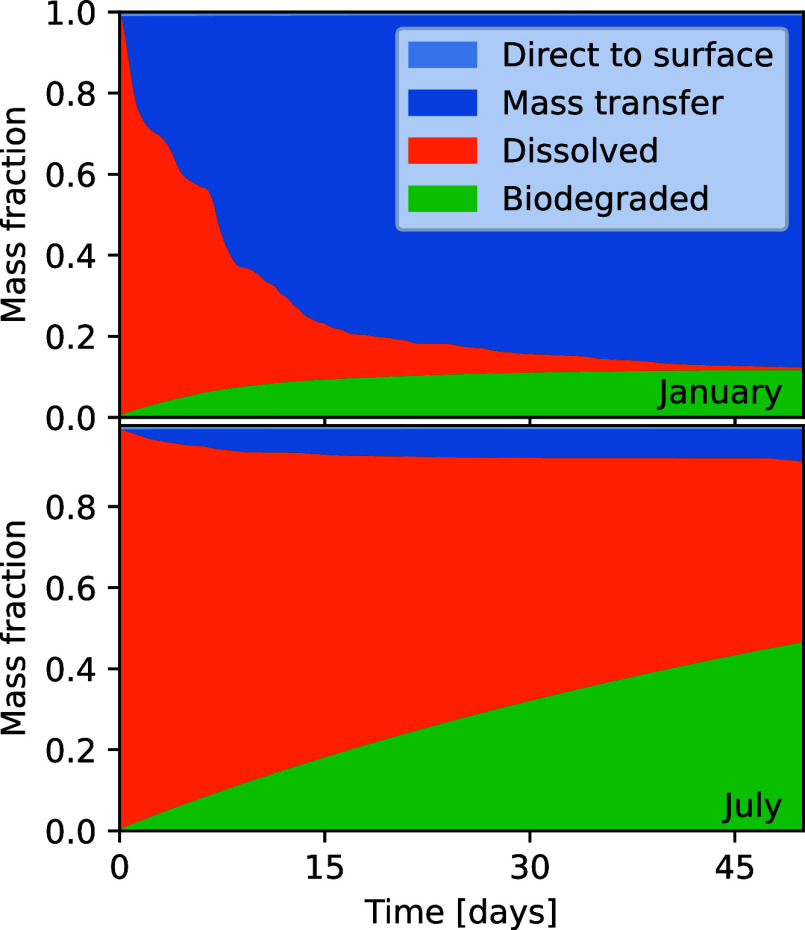
Mass balance as a function of time for a methane bubble released from 65 m depth at Station 1, for simulations starting on the first of the month in January and July. In both cases, about 1.2% of methane reaches the surface directly with the bubble.

Figure 6 shows how the mass balance develops over time for a simulation of Station 1, for a bubble released in January (top panel) and in July (bottom panel). In both cases, around 1% of the methane escapes directly to the atmosphere with the rising bubble (within minutes), while the rest dissolves. For the simulation that starts in January, the homogeneous water column (see Figure 3), and the correspondingly high eddy diffusivity leads to rapid vertical mixing of the dissolved methane and escape to the atmosphere via mass transfer. For the simulation starting in July, the dissolved methane largely remains trapped below the seasonal pycnocline, and escape to the atmosphere is much slower. Note that in both cases, the biodegradation half-life has (somewhat arbitrarily) been set to 50 days, yet we observe that the biodegraded amount is far smaller for the simulation starting in January, as more of the methane escapes to the atmosphere before biodegrading.

### 3.4 Diffusion-Reaction Model: Annual Averages

Figure 6 illustrates the strong effect of water column stratification on the fate of dissolved methane. Hence, to address the question of the overall fate of the methane from a continuous seep, we need to run an ensemble of simulations throughout the year and calculate the average fate. We have used environmental data from 2019 only, and let the simulations that run past the end of the year “wrap around” back to January. We run 36 simulations, evenly spaced throughout the year, and run each until the fraction of methane remaining dissolved in the water column is less than 10^–7^, such that essentially all methane is either biodegraded or has reached the atmosphere. We then calculate the average of the biodegraded fraction and the fraction escaped to the atmosphere across these 36 runs. We repeat the process for different values of the biodegradation half-life.

As mentioned, previously published biodegradation half-lives of methane in the marine environment vary considerably, from days^21−23^ to weeks^15^ and years.^21,24,25^ The results found in the current study are at the shorter end of this range, at 9–16 days. To reflect the uncertainty and to represent the large range of values found in the literature, we run the model for half-lives spanning from 1 day to 1000 days and show results as a function of half-life.

**Figure 7:**
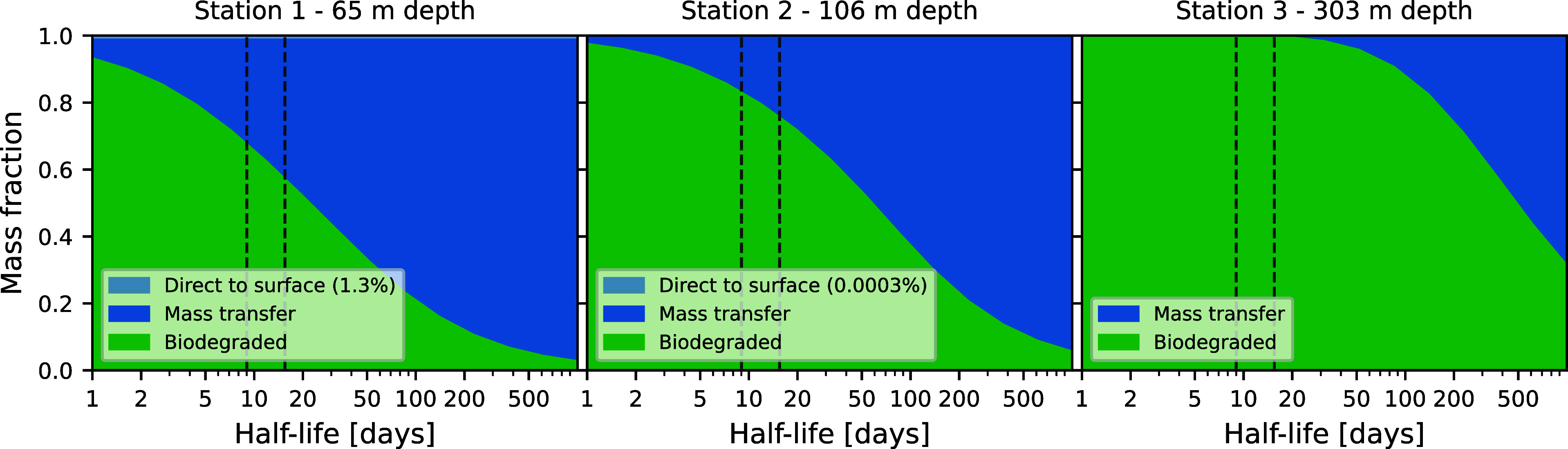
Average mass balance throughout the year as a function of biodegradation half-life. For each station, the results have been averaged over 36 model runs evenly spaced during the year, for each value of the biodegradation half-life. The simulations were run until the fraction of remaining methane in the water column is less than 10^–7^ of the released amount. The dashed lines indicate the 16–84 percentile range of half-lives found in Section 3.1. Note that the direct bubble transport is independent of half-life, as bubble rise happens on a time scale of minutes.

In Figure 7, we show the results of these simulations, indicating the split between direct bubble transport, mass transfer to the atmosphere, and biodegradation when averaged over a whole year and plotted as a function of biodegradation half-life.

We observe that for Stations 1 (65 m depth) and 2 (106 m depth), respectively, around 32–43% and 17–25% of the methane will escape to the atmosphere if the biodegradation half-life is in the range of 9 to 16 days, as found in Section 3.1. For the deeper release at Station 3 (303 m depth), however, the time for the dissolved methane to be mixed to the surface by the eddy diffusivity is sufficiently long that essentially all the methane will biodegrade in the water column, unless the biodegradation half-life is on the order of half a year.

### 3.5 Ventilation Rate

To further understand the relative importance of biodegradation and mass transfer to the atmosphere, we have calculated the normalized ventilation rate of dissolved methane from the water column to the atmosphere. We define this as *v* = *Ṁ*_atm_/*M*, where *M* is the mass of dissolved methane in the water column and *Ṁ*_atm_ is the rate at which methane escapes to the atmosphere. The normalized ventilation rate, *v*, has units of inverse time, just like the rate coefficient *k*_1_: They both describe the fraction of the mass that disappears per unit time. The fate of the dissolved methane is thus controlled by the ratio between *v* and *k*_1_, which determines the branching ratio between biodegradation and mass transfer to the atmosphere, as shown in Figure 1b.

**Figure 8:**
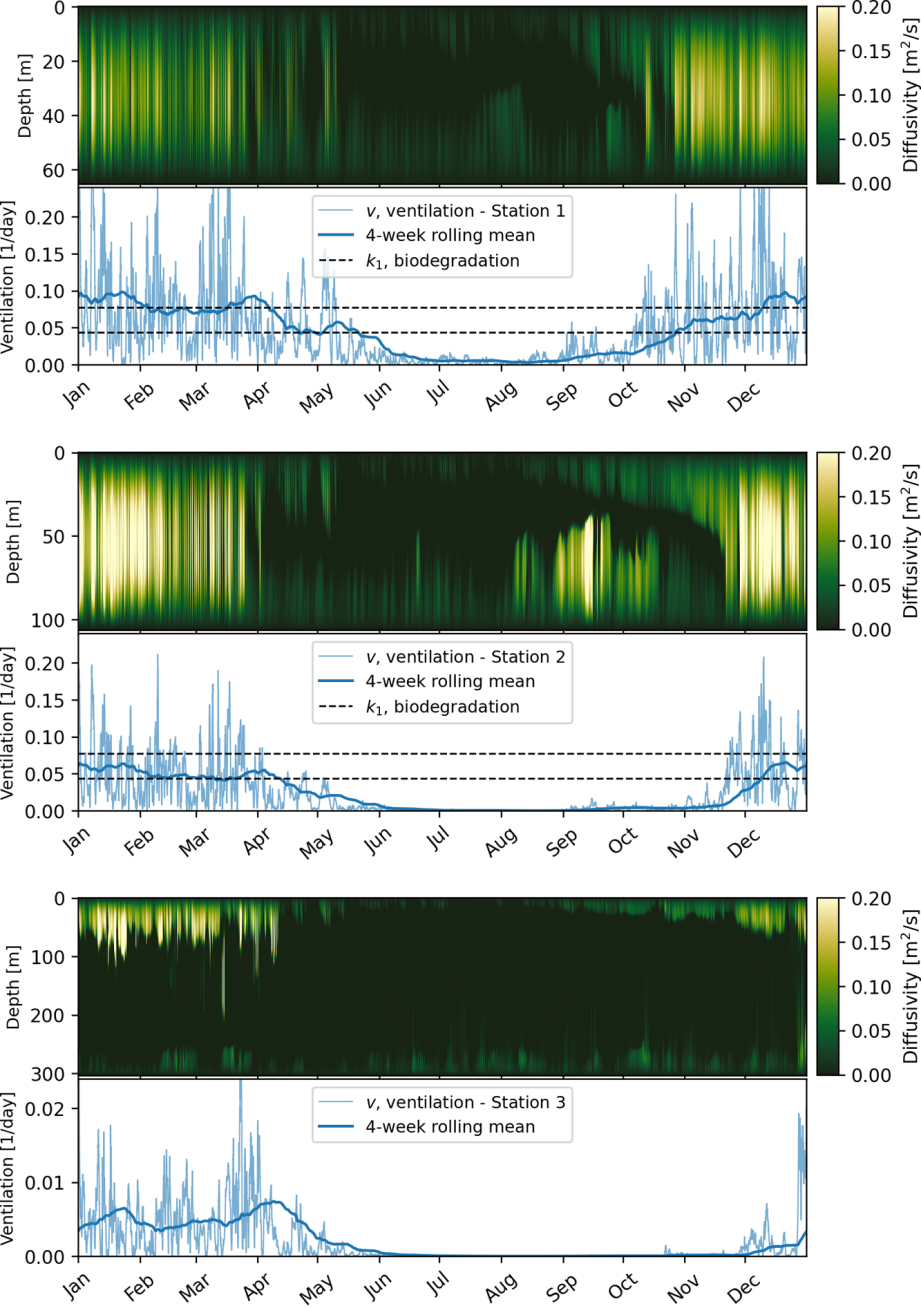
For each of the three stations: diffusivity profiles as a function of time (upper panels) and normalized ventilation rate, *v*, (lower panels). The diffusivity plot has been truncated to the range [0,0.2] to highlight the most relevant variations. First-order biodegradation rate coefficients, *k*_1_, corresponding to the 9–16 day (16–84 percentile) range of half-lives we found in Section 3.1 are also shown for comparison. Note that the range on the vertical axis is different for Station 3, to better show the ventilation rate, which is about an order of magnitude lower than for Stations 1 and 2.

In Figure 8, we present both the vertical eddy diffusivity and the ventilation rate as a function of time for all three stations. We clearly see that *v* is high when the diffusivity is high throughout the water column. The transport-inhibiting effect of the pycnocline is especially clearly seen in September and October for Station 2, when diffusivity is high both in the surface mixed layer and particularly in the deep interior, but low in between. The ventilation rate remains low until the water column becomes mixed through in late November.

In Figure 8, we also show the first-order biodegradation rate coefficients, *k*_1_, corresponding to the range of half-lives we found in Section 3.1. With these half-lives, biodegradation and ventilation rates are of the same order of magnitude at Stations 1 and 2 during the winter (November to April), while biodegradation dominates in the summer when there is little vertical mixing. For Station 3, which is much deeper and where there is at least weak stratification throughout the year, the ventilation rate is lower by an order of magnitude, and biodegradation is always dominant for the half-lives found in Section 3.1.

### 3.6 Uncertainties

Numerous assumptions and approximations have gone into the current study. The experimental work was carried out with water from a single location, the Trondheim fjord. Bacterial communities might be different in other locations, particularly in locations with active seeps. Furthermore, the experiments were carried out at relatively high methane concentrations compared with typical background values. We have assumed first-order kinetics, a commonly used approximation, but methane degradation rates are presumably also coupled to bacterial abundance.^79^

We use a water column model, assuming that horizontal transport will not significantly affect the biodegradation, vertical mixing, and mass transfer to the atmosphere. The diffusion-reaction equation is linear in concentration, which makes this a reasonable approximation, but horizontal mixing might lead to slower mass transfer rates if the dissolved methane is diluted to a point where the partial pressure of methane in the atmosphere becomes a relevant retarding factor. Effects like upwelling are also ignored and should be investigated in future studies with a full three-dimensional model. Furthermore, we have assumed clean-bubble mass transfer in the SBM, which will overestimate the fraction of methane that dissolves from the rising bubble and underestimate the direct bubble transfer to the surface.

### 3.7 Implications and Further Work

Seafloor seeps of methane can have significant impacts on climate change, and it is important to understand the processes that determine the mass balance of such seeps. Methane from seafloor seeps will typically dissolve in the water column, with only a small (or even zero) fraction of the methane reaching the surface directly with the bubbles.^6,8,17^

Previous studies have considered the fate of methane from seafloor seeps in the North Sea, but assumed biodegradation to be very slow^8^ or negligible,^6^ while other studies in, e.g., the Barents Sea have found faster biodegradation rates.^15^ Global methane budgets by, e.g., Etiope^80^ include published estimates for submarine seeps in depths up to 500 m, where “methane is expected to reach the atmosphere”. Clearly, such estimates will depend on what assumptions are made about the biodegradation rates. As previously mentioned, published half-lives range from days^21−23^ to years.^21,24,25^ We found rates in the range 9–16 days (see Sections 2.1 and 3.1), but it seems clear that more work is needed on this point.

In our modeling study, we have chosen to treat the biodegradation half-life, *t*_1/2_, as a free parameter, and present results as a function of *t*_1/2_ across a range from days to years, corresponding to the values reported in the literature.^21−25^ As shown in Figure 7, the fate of methane from seeps at the NCS can range from nearly all the methane reaching the atmosphere to all the methane biodegrading in the water column, depending on depth and biodegradation half-life.

Given the assumptions and approximations discussed in Section 3.6, our model results should not be interpreted as accurate predictions of the fate of methane from seeps for particular release locations, although we argue that it can give good estimates. Mainly, our model is a tool that can be used to explore the rate of methane release to the atmosphere under different conditions. As a water-column model, it is lightweight enough to permit, e.g., global sensitivity analysis.^81^ Hence, it can suggest which processes are most important in controlling the fate of released methane and point to the most important uncertainties that should be constrained by future research.

## Supporting Information Available

Additional results on Bayesian analysis; details on numerical diffusion-reaction solver; hydrography for stations 1–3; additional results for the bubble rise model (PDF)

## Footnotes

1 The model is available from https://github.com/socolofs/tamoc.

2 https://github.com/SINTEF/Fate-of-methane

3 https://github.com/SINTEF/Fate-of-methane
