# Supplementary material for: Fate of Dissolved Methane from Ocean Floor Seeps
Source: Environ Sci Technol. 2025 Apr 23;59(17):8516–26. doi: 10.1021/acs.est.5c03297 (PMC12060284; doi:10.1021/acs.est.5c03297)
Supplement: Supplementary file 1 — es5c03297_si_001.pdf [file es5c03297_si_001.pdf]

Supporting Information to "Fate of dissolved methane from ocean floor seeps"

Tor Nordam, Anusha L. Dissanayake, Odd Gunnar Brakstad, Sigrid Hakvåg,
Ida Beathe Øverjordet, Emma Litzler, Raymond Nepstad, Annika Drews, Johannes Röhrs

Prepared for *Environmental Science & Technology.*

Number of pages: 11
Number of figures: 8
Number of tables: 0

S1 Parameter estimation and Bayesian analysis

Estimation of optimal parameter values were performed based on maximum likelihood estimation (MLE) using the `lmfit` Python package, with the Nelder-Mead method. Initial values were set to $C_{tot} = 2.5$, $C_b = 1$, $\tau = 10$, with the latter constrained to the interval $[0.25, 100]$ (uniform prior), and the two former required to be non-negative but with no upper bound. The MCMC analysis (using `emcee`) started from the MLE results for the parameter values, using the uniform priors described in the manuscript text. A total of 10 000 steps were run using 100 walkers, and the first 1 000 were discarded (burn), while the remaining were thinned to every 10th step to obtain the final posterior samples, resulting in the values shown in the trace plot in Fig. S1 (produced with the `arviZ` Python package). All parameters were well constrained by the data to intervals narrower than indicated by the priors used, as can be seen in the corner plot in Fig. S2.

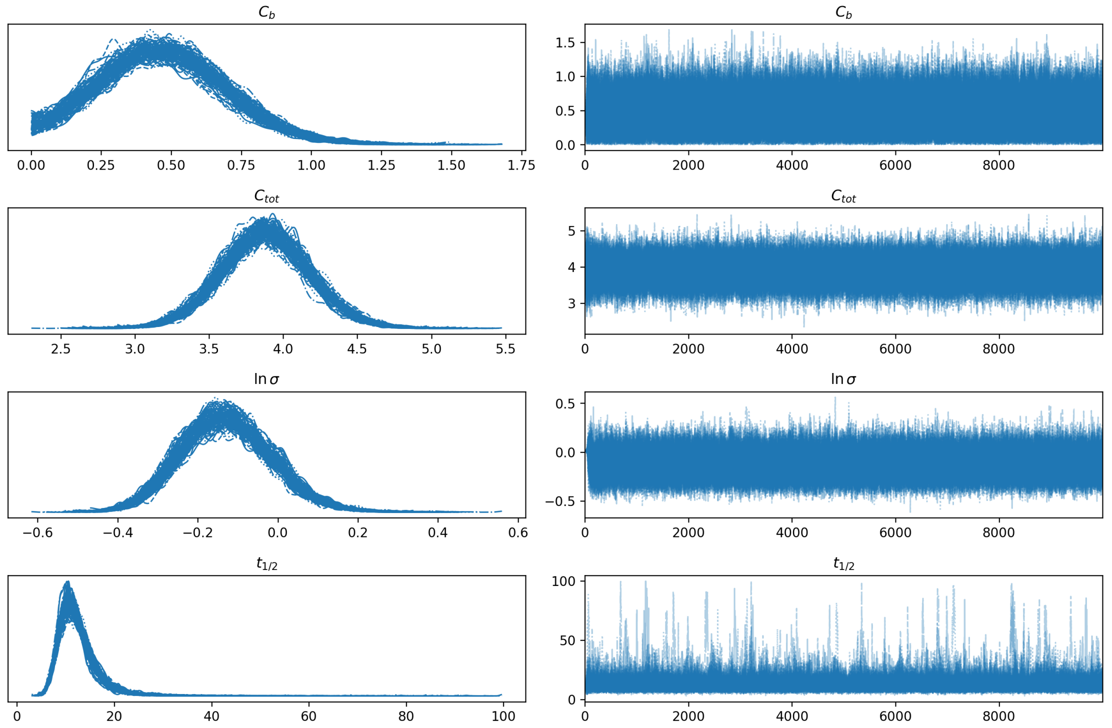

Figure S1: Trace plot of the MCMC walker samples for each parameter dimension.

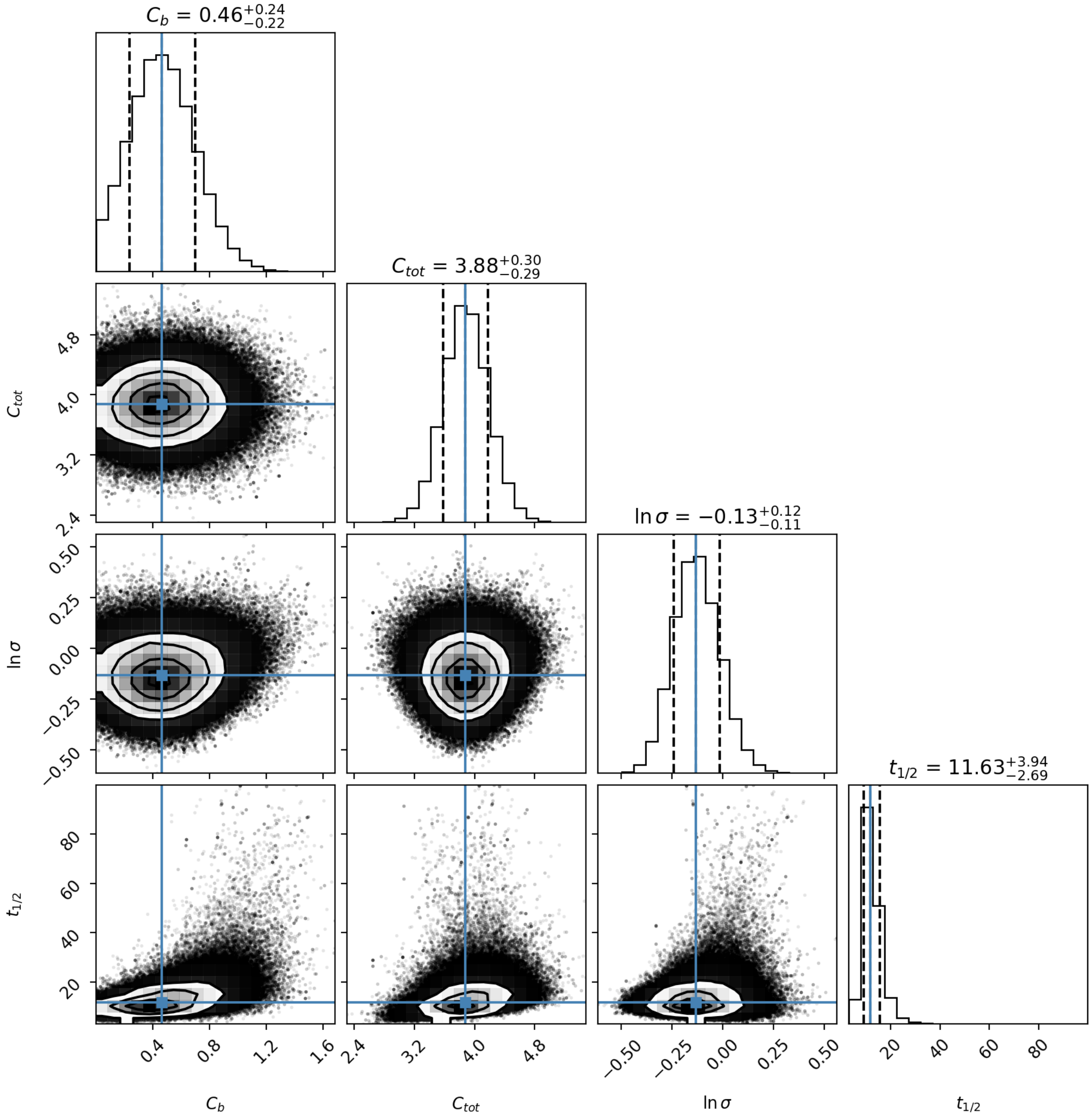

Figure S2: Corner plot of posterior from MCMC parameter estimation analysis, showing the 1D and 2D distributions of each parameter.

S2 Numerical implementation of the diffusion-reaction equation

The diffusion-reaction equation for a concentration $C(z,t)$ is

$$\frac{\partial C(z,t)}{\partial t} = \frac{\partial}{\partial z}\left(K(z)\frac{\partial C(z,t)}{\partial z}\right) + R\big(z, C(z,t)\big), \tag{1}$$

where $K(z)$ is the diffusivity. In our case, the reaction term corresponds to exponential decay of methane concentration, which means that $R(C) = -k_1 C$ where k_1 is the first-order biodegradation rate coefficient.

We use a finite-volume method to discretise Eq. (1), by dividing the z-axis into N cells. The cells have constant size Δz, with cell centers located at $z_j = (j + 1/2)\Delta z$, the surface at $z_{-1/2} = 0$ and the sea floor at $z_{N-1/2} = L$. We integrate over each cell, and use the divergence theorem to convert volume integrals over changing concentration to surface integrals over fluxes. By this approach, we obtain the following expression for the rate of change of average concentration in each cell:

$$\Delta z \frac{\mathrm{d}\overline{C}_j}{\mathrm{d}t} = \left(K\frac{\partial C}{\partial z}\right)_{j+\frac{1}{2}} - \left(K\frac{\partial C}{\partial z}\right)_{j-\frac{1}{2}} - \Delta z k_1 \overline{C}_j, \tag{2}$$

where the subscripts denote the z-position where the expressions are to be evaluated. We approximate the flux terms by (second order, central) finite difference approximation of the derivatives of C at the cell faces:

$$\Delta z \frac{\mathrm{d}\overline{C}_j}{\mathrm{d}t} = K_{j+\frac{1}{2}}\frac{C_{j+1} - C_j}{\Delta z} - K_{j-\frac{1}{2}}\frac{C_j - C_{j-1}}{\Delta z} - \Delta z k_1 \overline{C}_j. \tag{3}$$

At the boundaries, we need to eliminate the points outside the boundary from the equations. At the sea floor, we have a no-flux boundary condition, and hence we simply set the term representing the flux through the cell face at $z_{N-1/2}$ to zero, yielding

$$\Delta z \frac{\mathrm{d}\overline{C}_{N-1}}{\mathrm{d}t} = -K_{N-\frac{3}{2}}\frac{C_{N-1} - C_{N-2}}{\Delta z} - \Delta z k_1 \overline{C}_{N-1}. \tag{4}$$

At the sea surface, we have a mass transfer flux equal to $k_w C_0$ where k_w is the mass-transfer coefficient and C_0 is the concentration in the top cell. Just like above, we replace the relevant flux term with this expression, giving

$$\Delta z \frac{\mathrm{d}\overline{C}_0}{\mathrm{d}t} = K_{\frac{1}{2}}\frac{C_1 - C_0}{\Delta z} - k_w C_0 - \Delta z k_1 \overline{C}_0. \tag{5}$$

We discretise in time with the Crank-Nicolson scheme (implicit trapezoid, see, e.g., Gustafsson [2008, p. 39]), and introduce the short-hand notation $\alpha = \frac{1}{2}\frac{\Delta t}{\Delta z^2}$, $\beta = \frac{\Delta t}{2}$, and $C_n^i = C(z_n, t_i)$. At the sea surface boundary, we then have

$$\begin{aligned} &C_0^{i+1}\left(1 + \alpha K_{\frac{1}{2}} + \beta k_w + \beta k_1\right) + C_1^{i+1}\left(-\alpha K_{\frac{1}{2}}\right) \\ &\quad = C_0^i\left(1 - \alpha K_{\frac{1}{2}} - \beta k_w - \beta k_1\right) + C_1^i\left(\alpha K_{\frac{1}{2}}\right). \end{aligned} \tag{6}$$

For the interior points, we have

$$\begin{aligned} &C_{n-1}^{i+1}\left(-\alpha K_{n-\frac{1}{2}}\right) + C_n^{i+1}\left(1 + \alpha K_{n+\frac{1}{2}} + \alpha K_{n-\frac{1}{2}} + \beta k_1\right) + C_{n+1}^{i+1}\left(-\alpha K_{n+\frac{1}{2}}\right) \\ &\quad = C_{n-1}^i\left(\alpha K_{n-\frac{1}{2}}\right) + C_n^i\left(1 - \alpha K_{n+\frac{1}{2}} - \alpha K_{n-\frac{1}{2}} - \beta k_1\right) + C_{n+1}^i\left(\alpha K_{n+\frac{1}{2}}\right). \end{aligned} \tag{7}$$

And finally for the boundary at the sea floor:

$$
\begin{aligned}
&C_{N-2}^{i+1}\left(-\alpha K_{N-\frac{3}{2}}\right) + C_{N-1}^{i+1}\left(1 + \alpha K_{N-\frac{1}{2}} + \alpha K_{N-\frac{3}{2}} + \beta k_1\right) \\
&= C_{N-2}^{i}\left(\alpha K_{N-\frac{3}{2}}\right) + C_{N-1}^{i}\left(1 - \alpha K_{N-\frac{1}{2}} - \alpha K_{N-\frac{3}{2}} - \beta k_1\right).
\end{aligned} \tag{8}
$$

We can rewrite Eqs. (6)–(8) into a linear set of N equations

$$
\mathbf{L}\mathbf{C}^{i+1} = \mathbf{R}\mathbf{C}^{i}, \tag{9}
$$

where $\mathbf{C}^i = [C_0^i, C_1^i, \ldots, C_{N-1}^i]^T$ is the vector of concentrations at time t_i, etc. The matrices $\mathbf{L}$ and $\mathbf{R}$ are both tridiagonal, and remain constant in time. Hence, the system can be solved efficiently with the tri-diagonal matrix method (TDMA, see, e.g., Press et al. [2007, pp. 56–57]).

S3 Additional water column data for the three stations

In Figs. S3, S4, and S5 we show temperature, salinity and diffusivity for all three stations, for the year 2019. All three variables are plotted from the output of GOTM, but temperature and salinity are by design very similar to the temperature and salinity profiles from NorShelfRöhrs et al. [2018] that were provided as input to GOTM. The reason for this is that GOTM was run with a relaxation towards the input profiles, in order to produce high-resolution diffusivity data that are consistent with the provided hydrography.

For all cases, GOTM was run with a vertical resolution of 0.25 m and a timestep of 600 s. Simulations were started on December 25, 2018, and run until Januray 1, 2020, but only data for 2019 was used as input to the diffusion-reaction model.

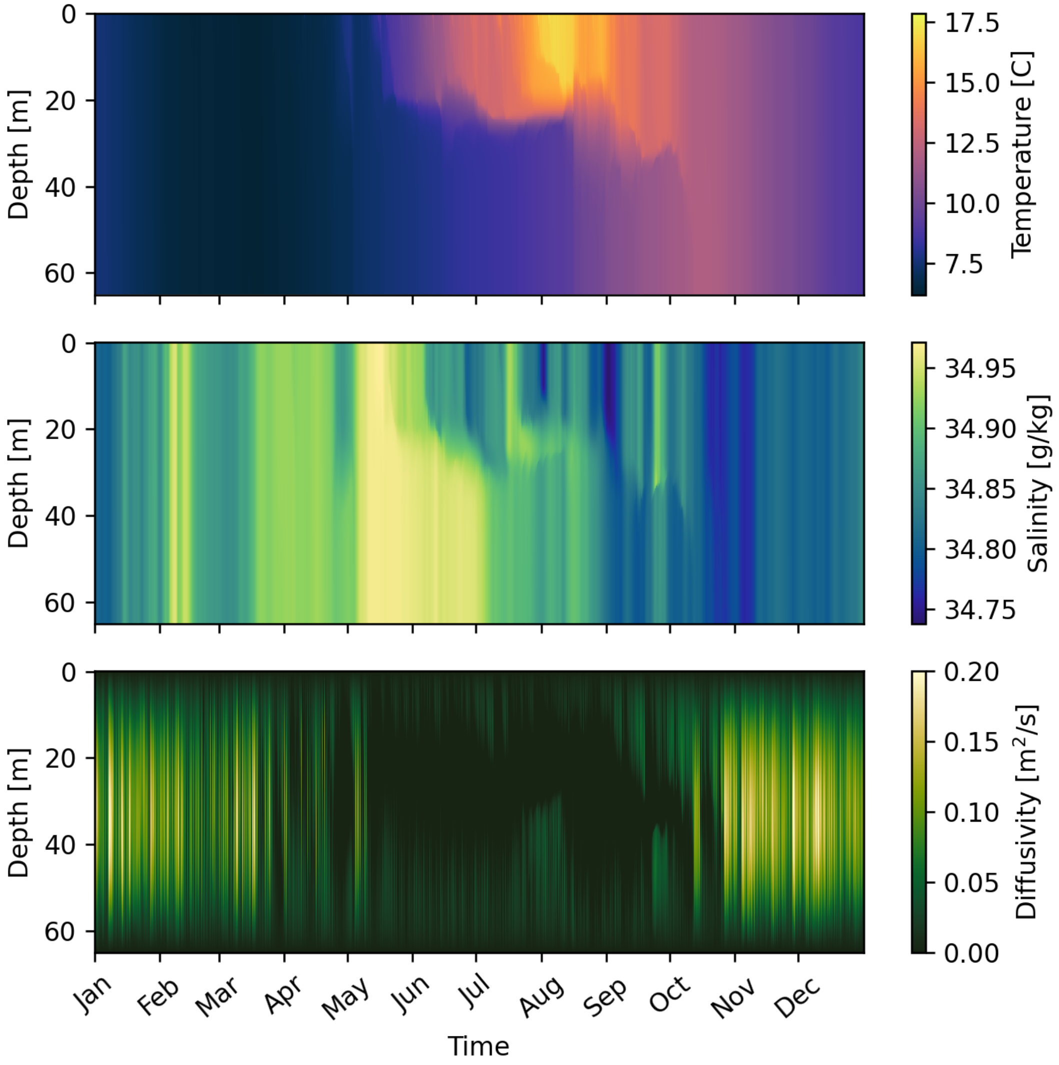

Figure S3: Temperature, salinity and eddy diffusivity for Station 1 (65 m depth), for the year 2019.

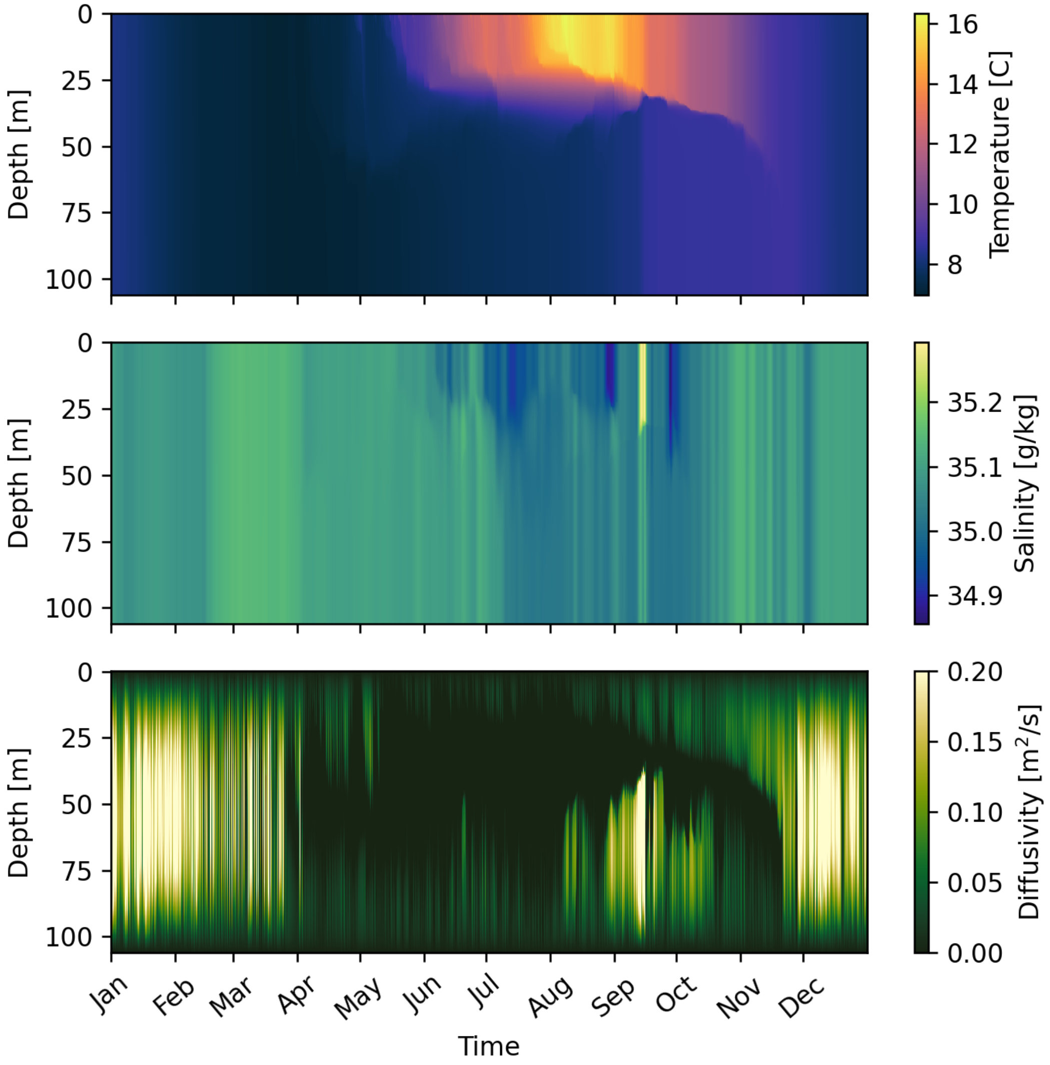

Figure S4: Temperature, salinity and eddy diffusivity for Station 2 (106 m depth), for the year 2019.

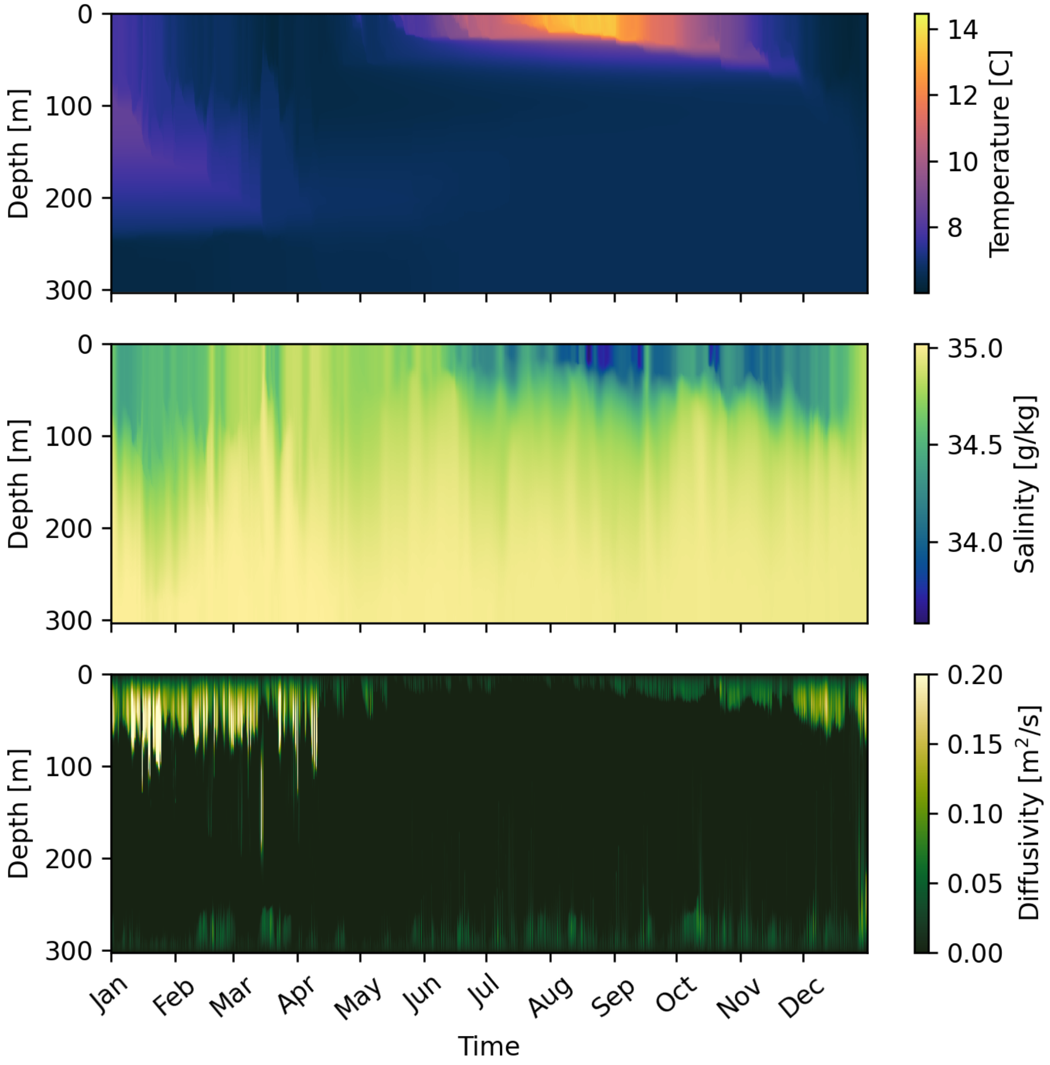

Figure S5: Temperature, salinity and eddy diffusivity for Station 3 (303 m depth), for the year 2019.

S4 Additional results from the Single Bubble Model

In Figs. S6, S7, and S8 we show output from the TAMOC Single Bubble Mobel (SBM) for each of the three stations. We observe that for Stations 1 and 2, the bubbles are predicted to reach the surface, but with only a very small fraction of the original methane remaining. At the surface, the bubbles will contain large fractions of nitrogen and oxygen taken up from the water column. Additionally, we note that even though the bubble at Station 1 is nearly as large at the surface as it was at depth, the far lower pressure means that it contains a smaller mass of gas. These results are in line with other published studies of seeping methane bubbles, for example von Deimling et al. [2011].

For Station 3, we find that the bubble dissolves completely, within about 80 m from the sea floor. This seems reasonably aligned with earlier published studies of methane flares by acoustic methods, where the signal from the bubbles dissappears after some distance from the sea floor (see, *e.g.*, McGinnis et al. [2006], Gentz et al. [2014]). We note that a direct comparison is not feasible, as those studies were conducted at different depths and in locations with possibly different hydrography and other ambient conditions (*e.g.* dissolved gases).

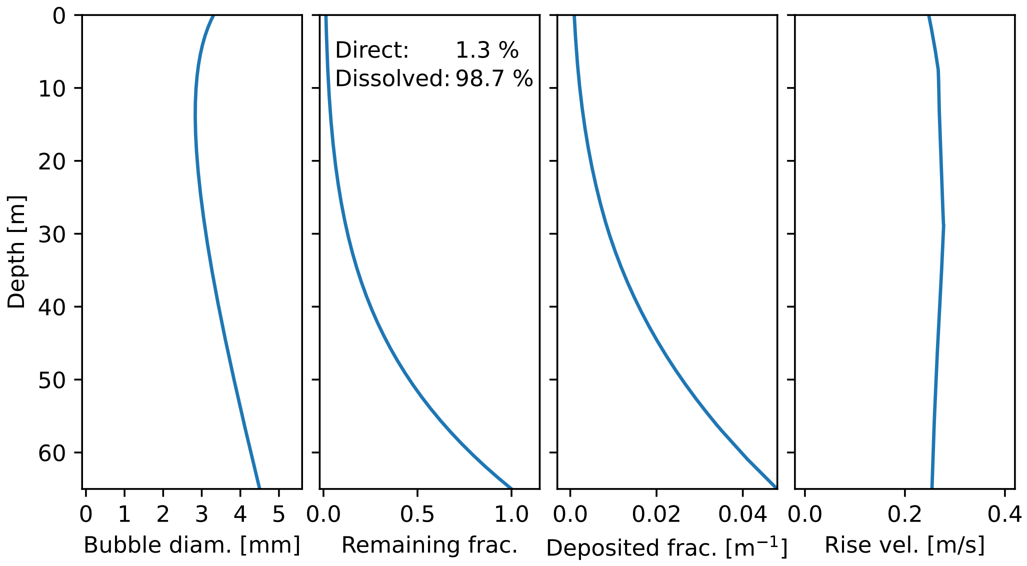

Figure S6: Results of running the SBM for Station 1, showing bubble size, remaining fraction of methane in the bubble, deposited fraction of methane per meter of water column, and rise velocity. When the bubble reaches the surface, 1.3% of the original methane remains, while 98.7% has been dissolved in the water column.

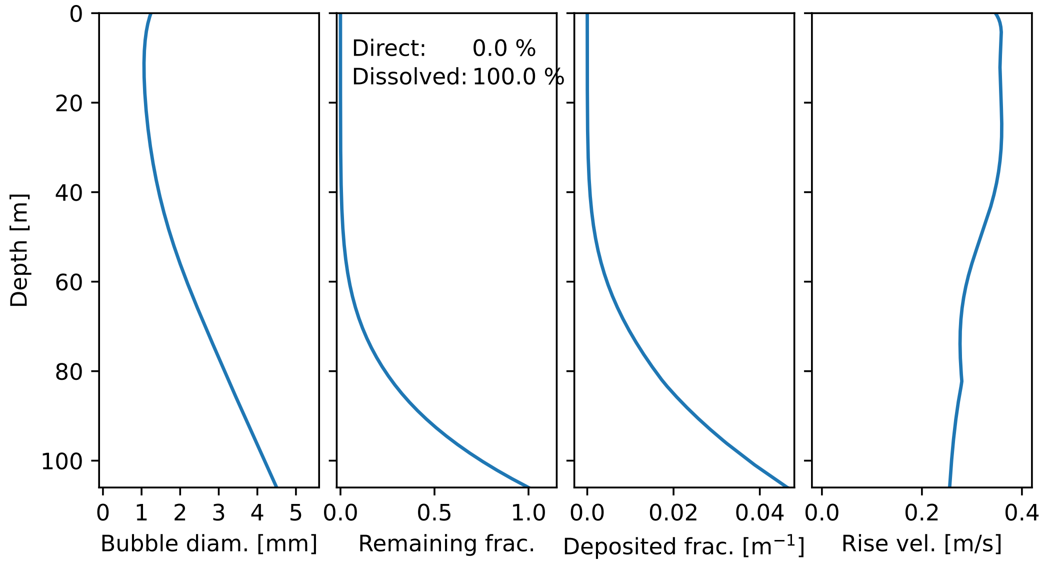

Figure S7: Results of running the SBM for Station 2, showing bubble size, remaining fraction of methane in the bubble, deposited fraction of methane per meter of water column, and rise velocity. When the bubble reaches the surface, around 0.0004% of the original methane remains, while the rest has been dissolved in the water column.

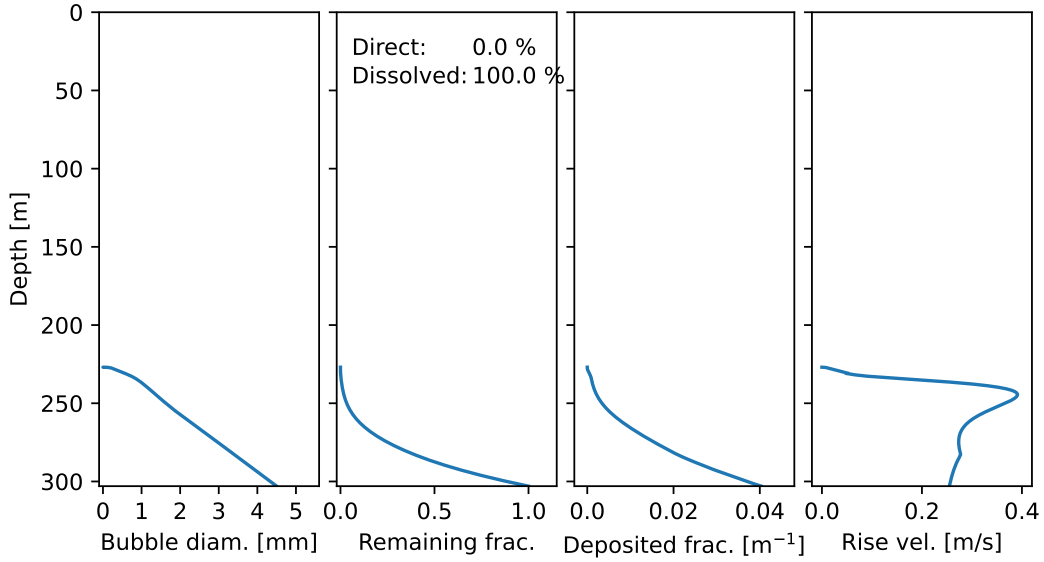

Figure S8: Results of running the SBM for Station 3, showing bubble size, remaining fraction of methane in the bubble, deposited fraction of methane per meter of water column, and rise velocity. In this case, the bubble dissolves completely before reaching the surface.

References

Torben Gentz, Ellen Damm, Jens Schneider von Deimling, Susan Mau, Daniel Frank McGinnis, and Michael Schlüter. A water column study of methane around gas flares located at the west spitsbergen continental margin. *Continental Shelf Research*, 72:107–118, 2014.

Bertil Gustafsson. *High order difference methods for time dependent PDE*. Springer-Verlag, Berlin Heidelberg, 2008.

DF McGinnis, Jens Greinert, Yu Artemov, SE Beaubien, and A Wüest. Fate of rising methane bubbles in stratified waters: How much methane reaches the atmosphere? *Journal of Geophysical Research: Oceans (1978–2012)*, 111(C9), 2006.

William H. Press, Saul A. Teukolsky, William T. Vetterling, and Brian P. Flannery. *Numerical Recipes*. Cambridge university press, New York, 3rd edition, 2007.

Johannes Röhrs, Ann Kristin Sperrevik, and Kai H. Christensen. NorShelf: A reanalysis and data-assimilative forecast model for the Norwegian Shelf Sea. Technical Report 04/2018, Norwegian Meteorological Institute, 2018.

J Schneider von Deimling, Gregor Rehder, Jens Greinert, DF McGinnnis, Antje Boetius, and Peter Linke. Quantification of seep-related methane gas emissions at tommeliten, north sea. *Continental Shelf Research*, 31(7-8):867–878, 2011.
